# Refractory cutaneo-gastric conduit fistula after esophagectomy repaired by a pectoralis major muscle flap and split-thickness skin graft

**DOI:** 10.1186/s40792-019-0672-z

**Published:** 2019-07-17

**Authors:** Rintaro Yoshida, Noriaki Sadanaga, Takuya Honboh, Hisashi Migita, Hiroshi Matsuura

**Affiliations:** 1 Department of Surgery, Saiseikai Fukuoka General Hospital, 1-3-46 Tenjin, Chuo-ku, Fukuoka, 810-0001 Japan; 2 Department of Plastic Surgery, Saiseikai Fukuoka General Hospital, 1-3-46 Tenjin, Chuo-ku, Fukuoka, 810-0001 Japan

**Keywords:** Gastric conduit ulcer, Skin fistula, Pectoralis major muscle flap, Split-thickness skin graft, Antesternal route

## Abstract

**Background:**

Gastric conduit ulcer after esophagectomy is not uncommon. In cases where a gastric conduit ulcer penetrates the adjacent organs, it is difficult to select a suitable treatment strategy. The treatment depends on the adjacent organs penetrated.

**Case presentation:**

We report a case in which a reconstructed gastric conduit ulcer penetrated the precordial skin in a patient who had undergone esophagectomy due to spontaneous esophageal rupture 28 years previously. To treat the cutaneo-gastric conduit fistula, we resected the fistula, covered the site of anastomosis with a major pectoralis muscle flap, and applied a split-thickness skin graft to the skin defect.

**Conclusions:**

In cases of gastric conduit trouble in patients treated via the antesternal route, a major pectoralis muscle flap is useful because of its rich blood supply and easy mobilization. In addition, a split-thickness skin graft should be applied to the skin defect.

## Background

The stomach is the preferred conduit after esophageal resection. Gastric conduit ulcers after esophagectomy are not uncommon, and it is difficult to select a suitable treatment strategy when the ulcer penetrates any of the adjacent organs. Treatment should be individualized to each case. We herein report a case of refractory cutaneous penetration by a gastric conduit ulcer that was successfully treated with resection of the fistula and transplantation of a pectoral major muscle flap and split-thickness graft, in a patient who had previously undergone esophagectomy due to spontaneous esophageal rupture.

## Case presentation

**Fig. 1:**
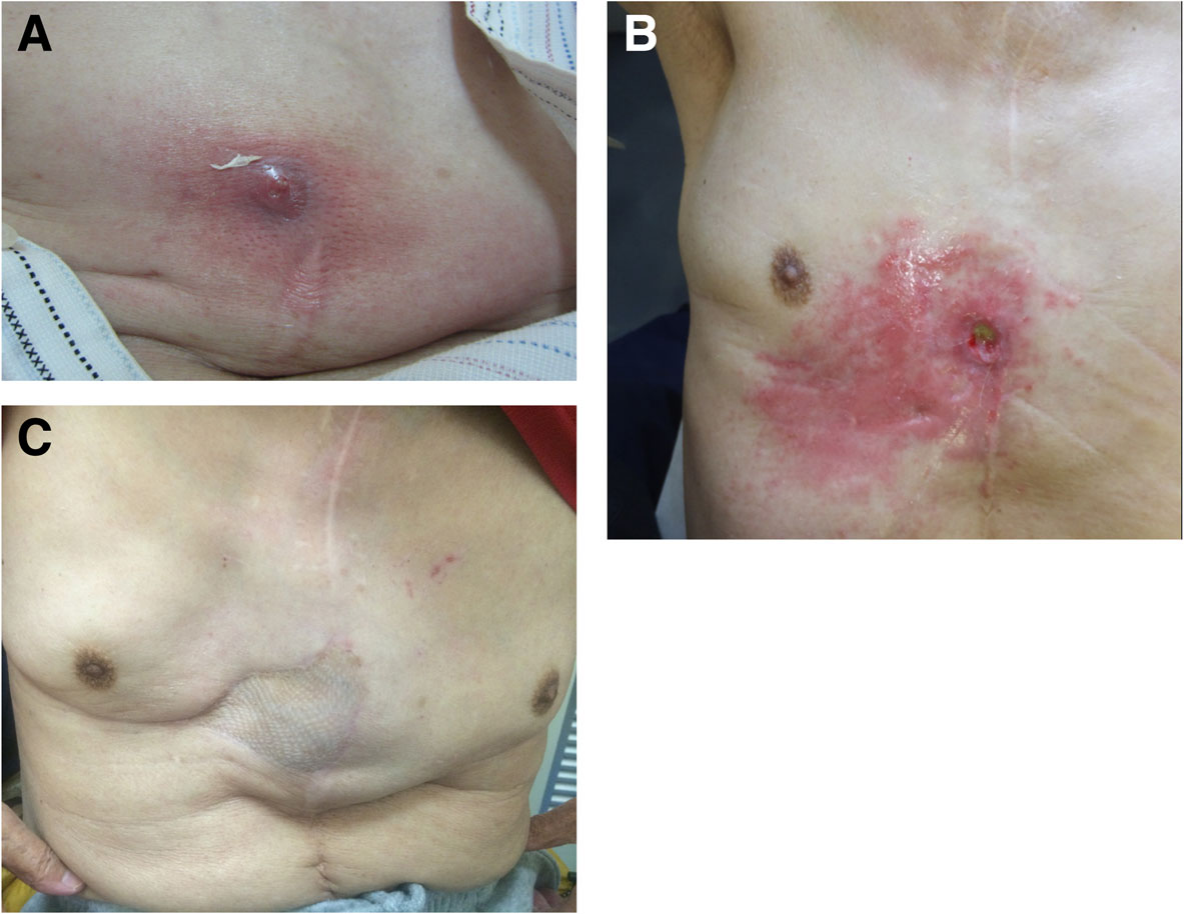
Photographs of the anterior chest. **a** The findings before the initial treatment. **b** The findings before surgery. **c** The findings at 2 years after surgery

A 70-year-old Japanese man, who had undergone esophagectomy with gastric conduit reconstruction via the antesternal route for spontaneous esophageal rupture 28 years previously, was hospitalized due to redness of the precordial skin, which had persisted for 1 month (Fig. 1a). Gastric juice was discharged from the gastric conduit through the skin fistula. He had taken low-dose aspirin due to a past history of left carotid artery obstruction and stenosis of the right carotid artery but had not taken proton pump inhibitors (PPIs). Laboratory studies showed mild inflammatory findings (WBC 9200/ul, C-reactive protein 5.7 mg/dl). No evidence of *Helicobacter pylori* infection was seen.

**Fig. 2:**
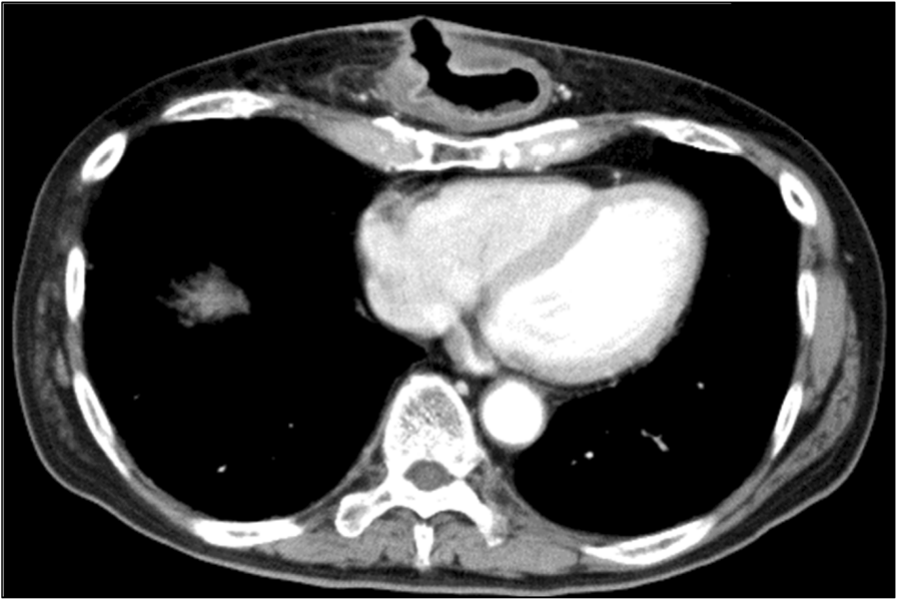
Chest CT showed a fistula between the gastric conduit and precordial skin

**Fig. 3:**
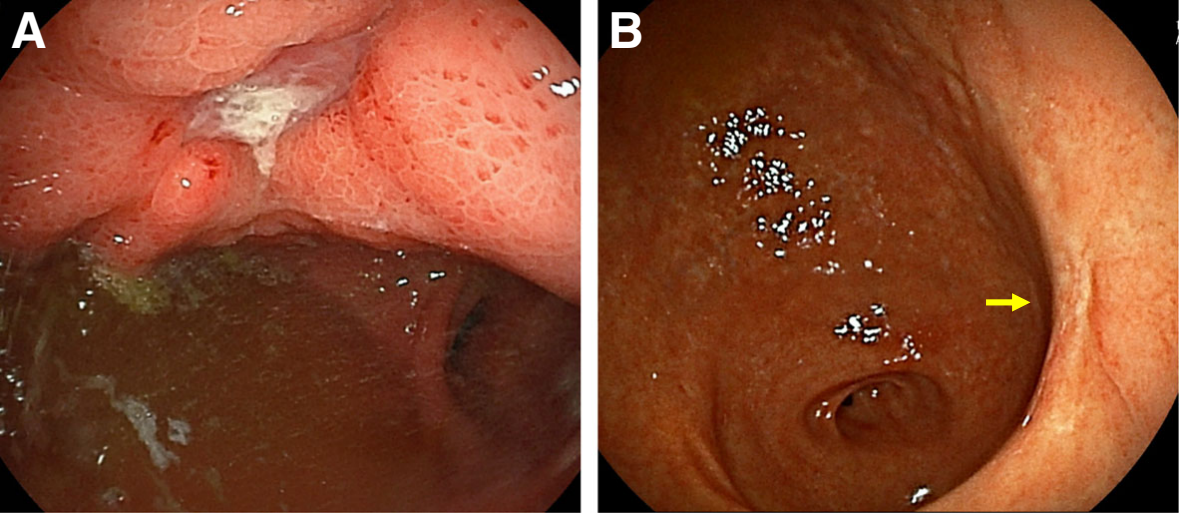
**a** Upper gastrointestinal fiberscopy on day 14 showed that the ulcer was located at the anterior wall of the middle gastric conduit. **b** At a follow-up examination performed 2 years after surgery, upper gastrointestinal fiberscopy showed an ulcer scar (arrow)

Computed tomography (CT) revealed a fistula between the thickened gastric conduit and skin (Fig. 2). He was diagnosed with a gastric conduit ulcer that penetrated through to the chest wall and was managed conservatively with fasting, the administration of a PPI and antibiotics, and continuous compression of the gastric conduit over the skin with a cotton ball. Upper gastrointestinal fiberscopy on day 14 revealed the ulcer was located at the anterior wall of the middle gastric conduit (Fig. 3a). An esophageal biopsy revealed no evidence of malignancy.

The fistula healed with conservative management. Although the fistula relapsed twice on the 37th and 58th days, respectively, it healed with conservative management.

On the 174th day from the onset, discharge was recognized again. A fistula of 1.5 cm in diameter was observed on the operation scar of the precordium at the nipple line, from which the gastric mucosa was seen. The skin around the fistula was reddish (Fig. 1b). On the 196th day, when the infection was completely controlled, resection of the refractory cutaneous fistula was performed with the transfer of a pectoralis major muscle pedicle flap and a split-thickness skin graft.

**Fig. 4:**
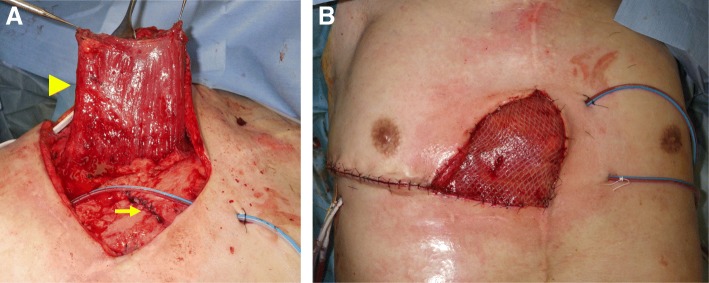
Operative findings. **a** The wall defect of the gastric conduit was closed with sutures (arrow) and a right pectoralis major muscle pedicle flap was freed (arrowhead). **b** The muscle pedicle flap was covered with a split-thickness skin graft

First, a skin incision of 3 cm × 2 cm in size was made on the fistula. The area around the fistula was debrided and the gastric conduit was partially resected together with the fistula. The wall defect was closed with interrupted layer-to-layer anastomosis (3-0 PDS®). Then, the tissue defect resulting from the resection was filled with a right pectoralis major muscle pedicle flap with the 2nd to 4th penetrating branches of the internal thoracic artery as a vascular pedicle. Finally, the muscle pedicle flap was covered with a split-thickness skin graft harvested from the left thigh (Fig. 4).

The patient had an uncomplicated postoperative course and was discharged on the 36th post-operative day. He has been free from relapse for 2 years (Fig. 1c).

## Discussion

The increasing use of the stomach as a conduit has led to increasing reports of peptic ulcer in the conduit. The risk of ulcer development in a gastric conduit is higher than that in the normal stomach [1]. The incidence of gastric conduit ulcer is reported to be 6.1–19.4% [2, 3]. The denervated gastric conduit recovers intraluminal acidity with time [4]. More than 80% of patients with a gastric conduit develop a peptic ulcer within 5 years. The time for the development of these ulcers varies widely, ranging from 1 month to as long as 150 months. The causes of gastric conduit ulcers remain controversial. Several mechanisms for the formation of gastric conduit ulcers have been hypothesized, including hypersecretion of gastric juice, *Helicobacter pylori* infection, delayed gastric emptying as a result of vagal denervation, bile juice regurgitation, insufficient blood supply due to gastric conduit creation, radiation, and the use of non-steroidal anti-inflammatory drugs (NSAIDs), aspirin, or steroids. Vagotomy, including the afferent sympathetic nerve fibers of 7th to 9th thoracic spinal cord, which transmits pain originating from the stomach, may be one of the reasons for the absence of symptoms. Consequently, the gastric conduit ulcer may penetrate any adjacent organ. Cutaneous fistulation after esophageal reconstruction is uncommon and has been reported to occur due to various mechanisms, such as leakage and radiation therapy [5]. It occurs regardless of whether the reconstruction route is antesternal or retrosternal. In our case, the use of aspirin without a PPI may have caused the peptic ulcer of the gastric conduit. PPI treatment reduces the risk of peptic ulcer associated with the continuous use of low-dose aspirin [6]. Long-term PPI treatment may reduce the rate of complications, and patient education after esophagectomy is necessary to prevent the development of gastric conduit ulcer. The surrounding organs perforated by refractory anastomotic leakage or gastric conduit ulcer vary and include the lung, trachea-bronchus, aorta, pericardium, chest wall, thoracic cavity, mediastinum, and skin [7–11]. As there is no standard management of fistula, the location, size, and extent of the fistula determine the appropriate treatment method. Several methods for managing anastomotic leakage and fistula have been suggested, including conservative treatment and more aggressive treatments, such as stent insertion, endoclip, and repair with a free jejunal graft and vascularized pedicle flaps [12].

The application of fibrin glue to a gastrocutaneous fistula after gastrostomy tube removal has been reported; however, this was contraindicated in cases involving a fistulous orifice of > 1 cm or infection of the fistulous tract [13]. Endoclips are conventional and effective but are too small to treat larger defects and are inadequate for closing scarred and hardened fibrotic tissue. In contrast, the application of an over-the-scope clip (OTSC) is a relatively less invasive method that is newly designed for the management of bleeding, perforation, and fistula. As the OTSC has a greater compressive force, it can be applied to larger defects and more hardened tissues [14]. The successful application of an OTSC in the treatment of esophagocutaneous fistula due to Boerhaave’s syndrome was reported [15]. However, it is difficult to maneuver the OTSC well in the narrow gastrointestinal tract lumen with a tangential view. This is especially problematic in the case reconstructed via the antesternal route, as the tortuous reconstructed tract makes it more difficult to maneuver. Thus, in the present case, we decided not to apply an OTSC. The present case was indeed a worthy target of the abovementioned endoscopic treatments; however, it was more important to achieve the complete removal of the lesion because repetitive fistula formation at the same site due to gastric conduit ulcer was recognized in the present case. Although we planned to use free jejunal grafts in cases involving relatively large fistulas or in which the inflammatory range was too wide, the present case was successfully treated by partial resection alone.

Muscle flaps, including the pectoralis major, sternocleidomastoid muscle, deltopectoral muscle, latissimus dorsi muscle, and diaphragm can also be used in the treatment of refractory gastric conduit defects. The choice of which muscle to use for the repair depends on the location of the fistula. Pectoralis major muscle flaps have a rich blood supply and are useful in the neck and precordial region. As blocking of sialic leakage is very important for the engraftment of plombage tissue, a closed fistula should be covered with a muscle flap with a rich blood supply.

Morita reported that a pectoralis major muscle flap was useful for repairing anastomotic leakage with refractory cutaneous fistula after reconstruction via the antesternal route for esophageal cancer [16]. Trimming and repair of the leakage site were initially performed and the anastomotic site was then covered with a muscle flap. A muscle flap repair was indicated under similar conditions to our case of cutaneo-gastric conduit fistula. The reported advantages of pectoralis major muscle flaps include the readily available source of vascularized tissue and the fact that it can be easily harvested for use in the head and neck [17]. In addition, a split-thickness skin graft should be considered in cases involving wide skin defects. Sadanaga reported that an esophageal defect and a wide skin defect of the anterior chest wall were successfully treated with a split-thickness skin graft and a pectoralis major muscle flap [18].

## Conclusions

We described a rare case of a patient who developed refractory cutaneo-gastric conduit fistula after esophagectomy, which had been performed to treat spontaneous esophageal rupture 28 years previously. The patient was successfully treated using a pectoralis major muscle flap and a split-thickness skin graft.

## Data Availability

Data sharing is not applicable to this article, as no datasets were generated or analyzed during the current study.

## Abbreviations

CT: Computed tomography
NSAIDs: Non-steroidal anti-inflammatory drugs
PPI: Proton pump inhibitor
